# Chronic Pain Treatment: The Influence of Tricyclic Antidepressants on Serotonin Release and Uptake in Mast Cells

**DOI:** 10.1155/2013/340473

**Published:** 2013-04-18

**Authors:** Ilonka Ferjan, Metoda Lipnik-Štangelj

**Affiliations:** Department of Pharmacology and Experimental Toxicology, Faculty of Medicine, University of Ljubljana, Korytkova Ulica 2, 1000 Ljubljana, Slovenia

## Abstract

The involvement of serotonin (5-HT) in chronic pain mechanisms is established. 5-HT inhibits central painful stimuli, but recent data suggests that 5-HT could also enhance pain stimulus from the periphery, where mast cells play an important role. We aimed in our study to clarify the influence of selected tricyclic antidepressants (TCAs) on mast cell function: secretion, uptake, and reuptake of 5-HT, that could interfere with 5-HT levels and in this way contribute to the generation of pain. As an experimental model, we used isolated rat peritoneal mast cells and incubated them with selected TCAs (clomipramine, amitriptyline, doxepin, and imipramine) under different experimental conditions. 5-HT release, uptake, and reuptake were determined spectrofluorometrically. We showed that TCAs were able to inhibit 5-HT secretion from mast cells, as well as uptake of exogenous 5-HT and reuptake of secreted 5-HT back into mast cells. The effects of TCAs were concentration dependent; higher concentrations of TCAs inhibited the secretion of 5-HT induced by compound 48/80, whereas lower concentrations of TCAs inhibited 5-HT uptake. The most effective TCA was halogenated clomipramine. As TCAs are well introduced in chronic pain treatment, the insight into mechanisms of action is important for an understanding of their effect in various pain conditions.

## 1. Introduction

Chronic pain is a complex neurobiological phenomenon with a variety of factors contributing to peripheral and central pain-signaling mechanisms. A common underlying mechanism of chronic pain is the presence of inflammation at the site of the damaged or affected tissue which causes release of several inflammatory mediators such a prostaglandins, bradykinin, and histamine. These agents increase the sensitivity of primary sensory neurons to painful stimuli [1]. Strong activation by proinflammatory mediators also drives the opening of voltage-gated sodium channels (VGSCs) that are crucial for central and peripheral sensitization and the excitability of neurons in the central and peripheral nervous systems [2–4]. The release of proinflammatory and immunoactive substances initiates therefore local actions and can result in a more generalized response that leads to a chronic pain condition.

Besides peripheral sensory pathways, there are central inhibitory or facilitatory pathways where various neurotransmitters and signaling molecules can contribute to the generation and/or maintenance of central as well as peripheral painful stimuli [1]. Among them, serotonin (5-HT) plays a complex role. In the central nervous system, monoaminergic (noradrenaline and 5-HT) and opioidergic neurons from descending pathways are inhibitory for pain transmission; in neuropathic pain, persistent pain is thought to be principally due to activation of descending pain facilitatory pathways and deactivation of descending pain inhibitory pathways [5–9]. In the spinal cord, convergence of peripheral inputs and descending pathways occurs. Here, the inhibitory molecules such as gamma-aminobutyric acid (GABA), endogenous opioids, and monoamines control the transmission of noxious stimuli [10, 11].

On the contrary of the inhibitory effect of 5-HT on central painful stimuli, recent findings suggest that 5-HT might enhance a pain stimulus from the periphery. It has been found that the association between increased 5-HT levels and increased number of mast cells in patients with chronic abdominal pain [12–14]. A possible relationship between the number of mucosal mast cells and rectal sensitivity has also been demonstrated in humans [14]. There is also evidence of a significant increase in mast cell numbers in patients with intestinal bowel syndrome. Along with increased mast cell counts, there is support that mast cell numbers directly correlate with abdominal pain in those patients [15]. On the other hand, we have only limited data about the role of mast cells in the central nervous system in the occurrence of chronic pain. The precise role of the mast cell-derived 5-HT in the chronic pain mechanisms is therefore still unknown.

To date, selected antidepressants are considered as an essential component of the therapeutic strategy for treatment of different types of persistent pain like neuropathic pain, painful polyneuropathy [16, 17], postherpetic neuralgia [18, 19] as well as rheumatoid arthritis, ankylosing spondylitis [20], and fibromyalgia [21], although the exact mechanisms involved in these processes are not fully known (for review see [1]). The main mechanism of action of antidepressants involves reinforcement of the descending inhibitory pathways by increasing the amount of noradrenaline and 5-HT in the synaptic cleft at both supraspinal and spinal levels. Further studies have demonstrated a critical role of VGSCs in different types of chronic pain syndromes; in this sense, antidepressants with property of blocking sodium channel have been shown to be effective in suppression of persistent pain signal [1]. We found in our previous studies that some antidepressants are able to influence 5-HT secretion from the mast cells [22, 23]. Since the impact of the mast-cells derived 5-HT in the persistent pain might be important, we were interested in present work to clarify the influence of selected antidepressants on different processes, controlled by mast cells, like secretion, uptake, and reuptake that could interfere with 5-HT levels and therefore with the generation and/or maintenance of pain.

## 2. Materials and Methods

### 2.1. Materials

Serotonin, amitriptyline, doxepin, imipramine, and clomipramine were obtained from Sigma, Steinheim, Germany. Compound 48/80, concanavalin A, bovine serum albumin, glucose, Tris-HCl, and phthaldialdehyde (OPT) were also obtained from Sigma Chemicals, Steinheim, Germany. HEPES was purchased from Merck, Darmstadt, Germany, and Percoll was obtained from Amersham Biosciences, Uppsala, Sweden. All other chemicals were of analytical grade. Spectrofluorometry was carried out on the spectrofluorometer Shimadzu RF-1501.

### 2.2. Animals

Wistar rats (200–350 g) were obtained from our own breeding colony. They were maintained under constant environmental conditions, with an ambient temperature of 22 ± 1°C, a relative humidity of 55 ± 10%, and a natural regimen of light-dark cycle. The animals were kept in cages Ehret type 4 (Germany); bedding material was Lignocel 3/4. They received standard rodent diet Altormin (Germany) and have free access to food and water. We used two animals for each experiment. All animal procedures have been approved by the National Animal Ethical Committee of the Republic of Slovenia and were conducted in accordance with the European Convention for the Protection of Vertebrate Animals Used for Experimental and Other Scientific Purposes (ETS 123).

### 2.3. Isolation of Mast Cells

Rat peritoneal mast cells were isolated from peritoneal cavity as follows: 10 mL of buffered salt solution was injected into the peritoneal cavity, and then the abdomen was gently massaged for 1.5 min. Mixed rat peritoneal cells were suspended in buffered salt solution with the following composition (mmol/L): NaCl 134.0, KCl 4.7, MgSO_4_ 1.2, CaCl_2_ 1.0, Tris-HCl 12.5, bovine albumin 1 mg/mL, and pH 7.4. The cell suspension was then centrifuged at 220 g for 10 min, and supernatants discarded. The collected cells were resuspended in buffered salt solution and centrifuged at 220 g for 10 min. For the preparation of purified mast cells (>98%), the cells were transferred to a HEPES-buffered (32 mmol/L) Percoll solution. A gradient of Percoll was created by centrifugation at 21000 g for 30 min at 4°C. After the centrifugation, Percoll was removed by washing the mast cell fraction in buffered salt solution, and additional centrifugation of the fraction, containing mast cells.

### 2.4. Treatment of Mast Cells with TCAs

Mast cells were resuspended in buffered salt solution (pH = 7.2) having the following composition (mmol/L): Na_2_HPO_4_ 6.7, KH_2_PO_4_ 6.7, NaCl 137, KCl 2.7, CaCl_2_ 1.0, bovine albumin 0.5 mg/mL, and glucose 1 g/L. Each sample contains between 5.10^5^ and 2.10^6^ mast cells.

1. In the secretion experiments, mast cells were preincubated with different concentrations (10^−8^–10^−4^ mol/L) of selected TCAs (amitriptyline, doxepin, imipramine, and clomipramine) for 10 min and then incubated in the presence of compound 48/80 (0.1 *μ*g/mL) for additional 10 min.
2. In the uptake experiments, mast cells were incubated with 5-HT (250 ng/sample) for 10, 30, or 60 min. The experiments were performed at 37°C or at 0°C in the presence of extracellular Ca^2+^ ions (10^−3^ mol/L) or in Ca^2+^-free medium. In the next group of experiments, mast cells were preincubated with different concentrations (10^−8^–10^−4^ mol/L) of selected TCAs (amitriptyline, doxepin, imipramine, and clomipramine) for 10 min and then incubated with 5-HT (250 ng/sample) for additional 30 min.
3. In the reuptake experiments, mast cells were incubated with compound 48/80 (0.2 *μ*g/mL) for 10, 30, or 60 min. In the next set of experiments, mast cells were preincubated with different concentrations (10^−8^–10^−4^ mol/L) of selected TCAs (amitriptyline, doxepin, imipramine, and clomipramine) for 10 min and then incubated in the presence of compound 48/80 (0.2 *μ*g/mL) or concanavalin A (100.0 *μ*g/mL) for additional 60 min.

After the incubation, the secretion, uptake, or reuptake of 5-HT was stopped by cooling the tubes in an ice-cold bath.

### 2.5. Determination of 5-HT Secretion, Uptake, and Reuptake

5-HT was determined in the supernatants and in the cell fraction, using a spectrofluorometric method and omitting the extraction procedure (for details see [24]). Samples (1 mL) were warmed in the presence of 0.05 mL cysteine (3%), 1.1 mL HCl (37%), and 0.07 mL OPT (0.2%) at 75°C for 15 min. After that they were cooled in an ice-cold bath, and 5-HT was measured spectrofluorometrically at excitation wavelength 360 nm and emission wavelength 478 nm. 5-HT was determined in the supernatants and in the cell fraction. 5-HT release was expressed as a percentage of the total 5-HT in the sample. All values were corrected for spontaneous 5-HT release, which was always <7.0%.

### 2.6. Statistical Analyses

Determinations of 5-HT content are shown as means ± standard error of the mean (SEM) of five independent assays. For each treatment and controls, four samples were analyzed. Student's *t*-test was used for statistical analysis. For all tests, *P* < 0.05 was considered to be statistically significant.

## 3. Results

### 3.1. Inhibitory Effect of Antidepressants on 5-HT Release

**Figure 1:**
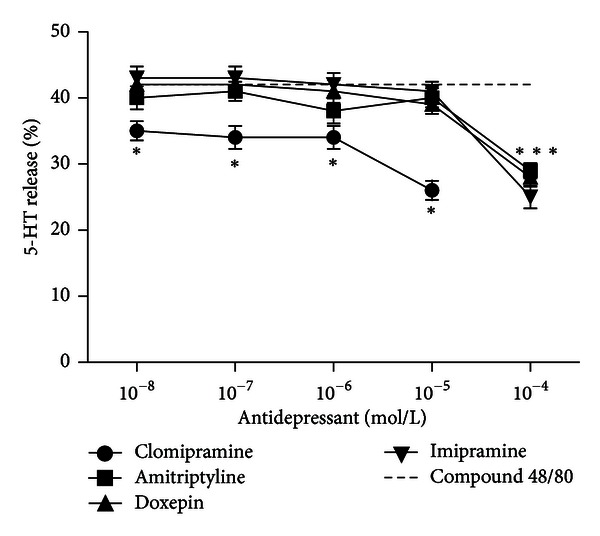
The influence of selected TCAs on 5-HT release from mast cells after stimulation of the cells with compound 48/80 (0.1 *μ*g/mL). Mast cells were preincubated with different concentrations (10^−8^–10^−4 ^mol/L) of antidepressants (amitriptyline, doxepin, imipramine, and clomipramine) for 10 min and then incubated with compound 48/80 for further 10 min. Results are expressed as a percentage of the total 5-HT in the sample. Each point represents mean ± SEM of 5 experiments. *P < 0.05 versus compound 48/80.

The secretagogue, compound 48/80, releases 5-HT from mast cells. After 10 min of incubation of mast cells with compound 48/80 (0.1 *μ*g/mL) 5-HT release is approximately 42%. The results show that TCAs are able to inhibit 5-HT secretion, induced by compound 48/80 from mast cells. The effect is dose dependent and occurs at higher concentrations of TCAs only. The inhibitory effect of TCAs depends on the polarity of the drug; the halogenated derivative clomipramine is significantly more potent than other used antidepressants (Figure 1).

### 3.2. The Effect of Antidepressants on 5-HT Uptake and Reuptake into Mast Cells

**Figure 2:**
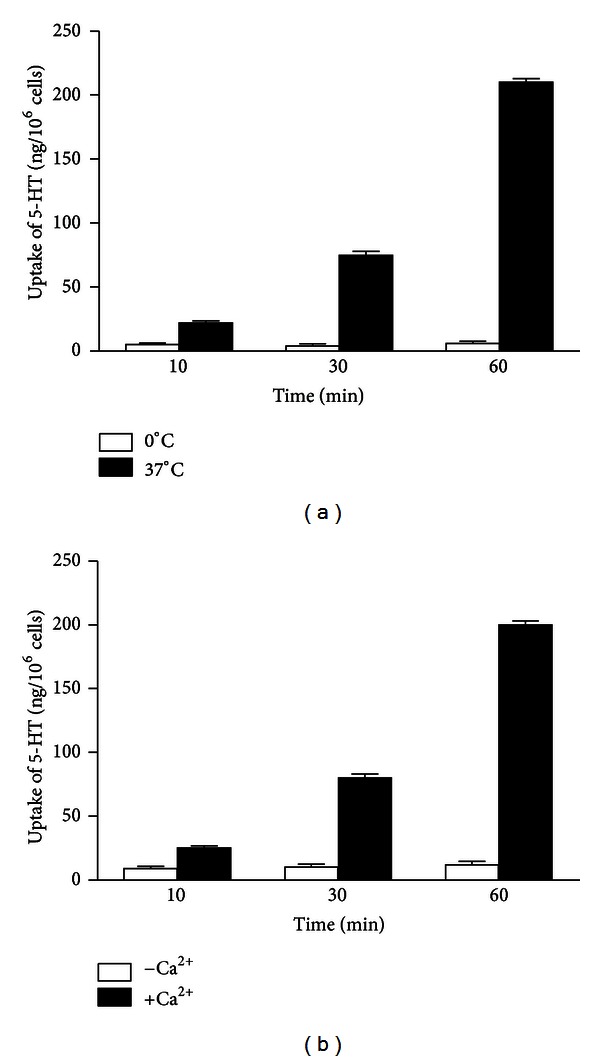
The effect of time of incubation on 5-HT uptake into mast cells. The mast cells were incubated with exogenous 5-HT (250 ng/sample) for 10, 30, or 60 min. (a) The effect of temperature of the medium on 5-HT uptake: mast cells were incubated with 5-HT at 37°C or at 0°C. (b) The effect of extracellular Ca^2+^ ions on 5-HT uptake: mast cells were incubated with 5-HT in the presence of extracellular Ca^2+^ ions (10^−3 ^mol/L) or in Ca^2+^-free medium. Each bar represents mean ± SEM of 5 experiments.

The results show that mast cells are capable to remove exogenous 5-HT from incubation medium. The uptake involves an active process which depends on temperature and time of incubation of mast cells with exogenous 5-HT. At 37°C it increases with time of incubation of mast cells with exogenous 5-HT, whereas at 0°C it is inhibited (Figure 2(a)). The uptake requires the presence of extracellular Ca^2+^ ions. In the medium, containing extracellular Ca^2+^ ions (10^−3 ^mol/L), the uptake increases with time of incubation. In contrast, the uptake is significantly inhibited in Ca^2+^-free medium (Figure 2(b)).

**Figure 3:**
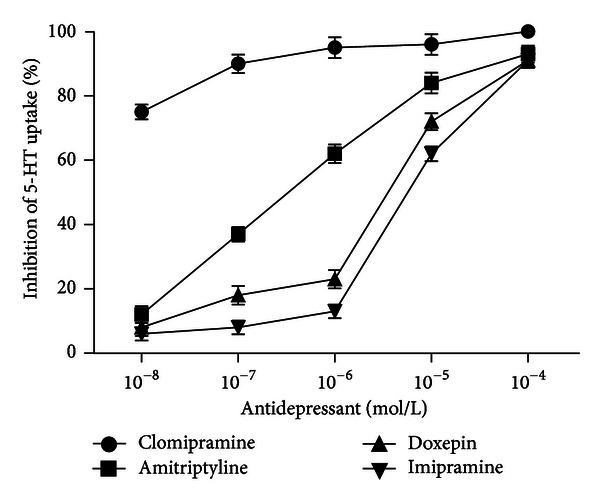
The influence of selected TCAs on the uptake of 5-HT into mast cells. Mast cells were preincubated with different concentrations (10^−8^–10^−4 ^mol/L) of antidepressants (amitriptyline, doxepin, imipramine, and clomipramine) for 10 min. After that, mast cells were incubated with exogenous 5-HT (250 ng/sample) for the next 30 min. Each point represents mean ± SEM of 5 experiments.

In the presence of extracellular Ca^2+^ ions (10^−3 ^mol/L), TCAs inhibit 5-HT uptake into mast cells in a dose-dependent manner. The most potent compound is halogenated antidepressant clomipramine, where inhibition of exogenous 5-HT uptake is observed at concentration 10^−8 ^mol/L (Figure 3).

**Figure 4:**
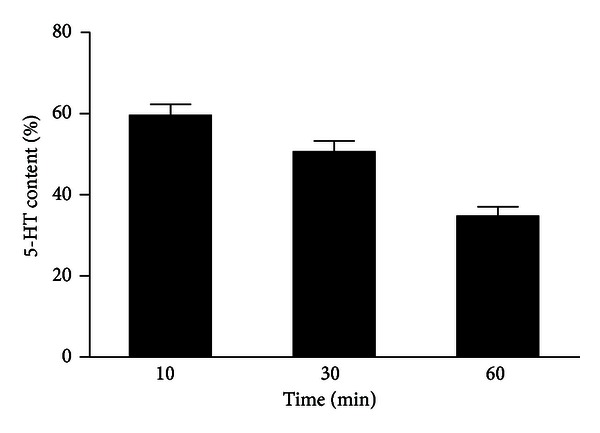
The effect of time of incubation on 5-HT content in the medium after stimulation of mast cells with compound 48/80. Mast cells were incubated with compound 48/80 (0.2 *μ*g/mL) for 10, 30, or 60 min. Each point represents mean ± SEM of 5 experiments.

In the next group of experiments, we demonstrated that mast cells are able to reuptake released 5-HT after stimulation of mast cells with compound 48/80. The reuptake is time dependent; after 10 min of incubation of mast cells with compound 48/80 (0.2 *μ*g/mL), it releases an average 60% of the total 5-HT. After 60 min of incubation, the amount of 5-HT was significantly reduced in comparison to 10 min incubation, which indicates that mast cells are capable to reuptake released 5-HT from the medium (Figure 4).

**Figure 5:**
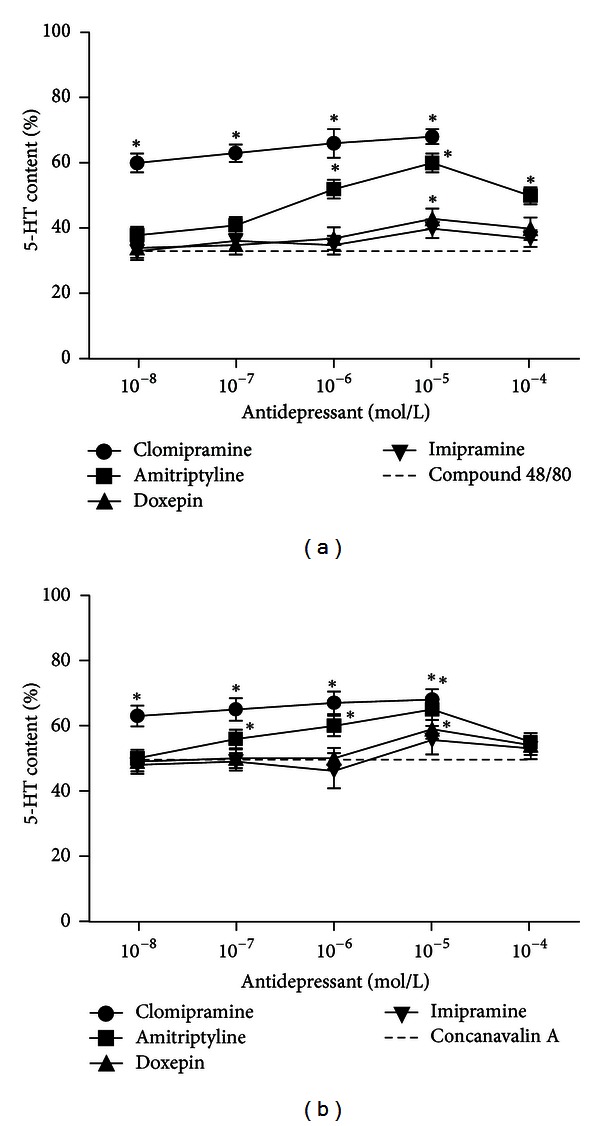
The influence of selected TCAs on 5-HT content after stimulation of mast cells with different secretagogues. (a) Mast cells were preincubated with increasing concentrations (10^−8^–10^−4 ^mol/L) of antidepressants (amitriptyline, doxepin, imipramine, and clomipramine) for 10 min and then incubated with compound 48/80 (0.2 *μ*g/mL) for the next 60 min. (b) Mast cells were preincubated with increasing concentrations (10^−8^–10^−4 ^mol/L) of antidepressants (amitriptyline, doxepin, imipramine, and clomipramine) for 10 min and then incubated with concanavalin A (100 *μ*g/mL) for the next 60 min. Each point represents mean ± SEM of 5 experiments. *P < 0.05 versus compound 48/80 (a) or concanavalin (b).

In further experiments, we examined the influence of selected TCAs on reuptake of 5-HT into mast cells after long-term (60 min) incubation of mast cells with different secretagogues, compound 48/80, and concanavalin A. Our results show that preincubation of mast cells with selected TCAs leads to inhibition of 5-HT reuptake into mast cells. The inhibition is dose dependent and differs between used TCAs; the most potent is halogenated antidepressant clomipramine. In Figure 5, we show that 60 min after the stimulation of mast cells by secretagogues (compound 48/80 and concanavalin A), the released 5-HT in the medium represents 36% and 49%, respectively, in comparison to the total 5-HT of the sample. The preincubation of mast cells with selected TCAs in concentration range from 10^−8^ to 10^−5 ^mol/L leads to inhibition of 5-HT reuptake into mast cells, in a dose-dependent manner. Therefore, after 60 min preincubation of mast cells with increasing concentrations of TCA, we observed higher concentrations of released 5-HT in the medium in comparison to the mast cell which have not been preincubated with TCA (Figure 5).

## 4. Discussion

Recent studies have indicated a strong communication between immune, endocrine, and nervous systems in the maintenance of chronic pain, where 5-HT plays significant role [25]. So far, we believed that 5-HT inhibited the generation of painful stimuli on the central nervous system level, but recent evidence indicates that 5-HT might be associated also by an increase pain transmission from the periphery, where mast cells play an important role [26, 27].

Using rat mast cells from peritoneal cavity, we show that TCAs influence mast cell-derived 5-HT levels via at least three different mechanisms: secretion of 5-HT, uptake of exogenous 5-HT, and reuptake of secreted 5-HT. At first, selected TCAs are able to inhibit the secretion of 5-HT from mast cells. The inhibition is dose dependent, and halogenated clomipramine has been found to be the most potent in comparison to imipramine, doxepin, and amitriptyline. The inhibition of 5-HT secretion from mast cells contributes to lower concentration of 5-HT at periphery and therefore could diminish sensitization of sensory nerve endings by 5-HT, which is important for the generation of peripheral painful stimuli [28, 29]. It is already known that approximately 95% of 5-HT in the body is produced in the peritoneal cavity, and inhibition of 5-HT secretion from mast cells might be beneficial in the treatment of chronic abdominal pain [12]. Our results support recent findings, where the association between enhanced mast cells number and 5-HT levels has been suggested in patients with chronic abdominal pain [14, 15]. With this regard, 5-HT has been proposed as an important mast cell mediator which could interact with peripheral nerves leading to increased sensitivity in the gut and chronic abdominal pain [30–33].

However, the precise role of mast cells in these cases has not been clarified yet, and several issues remain to be addressed. Beside 5-HT, mast cells release several mediators like histamine, tryptase, proteoglycans, leukotriene C4, platelet activating factor, and prostaglandin D2. All of them can activate sensory nerves, leading to visceral hyperalgesia/allodynia [29]. On the other hand, mast cells not only degranulate and release proinflammatory substances but also may be in closer proximity to the cholinergic nerves thereby altering GI motility and hypersensitivity (i.e., increased abdominal pain). The detection of abnormalities of 5-HT metabolism in the peritoneal cavity has therefore generated a particular interest [34–36].

In the central nervous system, 5-HT contributes to the inhibition of the pain signal transmission. In this process, serotonergic neurons from descending inhibitory pathways, and not mast cells, are crucial to derive 5-HT for synaptic transmission. It is already known that TCAs inhibit 5-HT uptake into serotonergic neurons and on this way enhance the concentration of 5-HT in synaptic cleft and inhibition of central painful stimuli. Moreover, the antidepressants with a property of blocking sodium channel (i.e., VGSCs) have been shown to be effective in suppression of persistent pain signal because these channels play a fundamental role in the excitability of neurons in the central and peripheral nervous system, as well [25]. In addition, we show in our study that TCAs are able to inhibit uptake of 5-HT into mast cells that could also contribute to higher concentrations of 5-HT in the central nervous system.

At the periphery, TCAs effects seem much more complex. They inhibit secretion of 5-HT from mast cells, which leads to diminished concentrations of 5-HT. In addition, they are also able to inhibit an uptake of exogenous 5-HT, as well as reuptake of secreted 5-HT from mast cells back into mast cells, which causes higher levels of 5-HT in the environment. In the peritoneal cavity, mast cells represent an important source of 5-HT, and when the secretion of 5-HT from mast cells is inhibited, the 5-HT-mediated sensitization of sensory might be inhibited as well.

## 5. Conclusions

In summary, we have found that TCAs are able to inhibit 5-HT secretion from mast cells, as well as uptake of exogenous 5-HT and reuptake of secreted 5-HT back into mast cells. All of these events influence 5-HT levels and as a consequence could contribute to a generation and maintenance of painful stimuli in the body. As TCAs are well established in the chronic pain treatment, the insight into their mechanisms of action is crucial for an understanding of their effects in various pain conditions. In this respect, our study provides a simple *in vitro* approach for the mechanistic studies of compounds, aimed for the modulation of 5-HT levels by mast cells.

## References

[1] Dharmshaktu P, Tayal V, Kalra BS (2012). Efficacy of antidepressants as analgesics: a review. *Journal of Clinical Pharmacology*.

[2] Berta T, Poirot O, Pertin M, Ji RR, Kellenberger S, Decosterd I (2008). Transcriptional and functional profiles of voltage-gated Na^+^ channels in injured and non-injured DRG neurons in the SNI model of neuropathic pain. *Molecular and Cellular Neuroscience*.

[3] Pertin M, Ji RR, Berta T (2005). Upregulation of the voltage-gated sodium channel *β*2 subunit in neuropathic pain models: characterization of expression in injured and non-injured primary sensory neurons. *Journal of Neuroscience*.

[4] Lai J, Porreca F, Hunter JC, Gold MS (2004). Voltage-gated sodium channels and hyperalgesia. *Annual Review of Pharmacology and Toxicology*.

[5] McWilliams LA, Cox BJ, Enns MW (2003). Mood and anxiety disorders associated with chronic pain: an examination in a nationally representative sample. *Pain*.

[6] McWilliams LA, Goodwin RD, Cox BJ (2004). Depression and anxiety associated with three pain conditions: results from a nationally representative sample. *Pain*.

[7] Von Korff M, Crane P, Lane M (2005). Chronic spinal pain and physical-mental comorbidity in the United States: results from the national comorbidity survey replication. *Pain*.

[8] Kirmayer LJ, Robbins JM, Dworkind M, Yaffe MJ (1993). Somatization and the recognition of depression and anxiety in primary care. *American Journal of Psychiatry*.

[9] Hudson JI, Hudson MS, Pliner LF (1985). Fibromyalgia and major affective disorder: a controlled phenomenology and family history study. *American Journal of Psychiatry*.

[10] Woolf CJ (1983). Evidence for a central component of post-injury pain hypersensitivity. *Nature*.

[11] Cardenas DD, Warms CA, Turner JA, Marshall H, Brooke MM, Loeser JD (2002). Efficacy of amitriptyline for relief of pain in spinal cord injury: results of a randomized controlled trial. *Pain*.

[12] Cremon C, Carini G, Wang B (2011). Intestinal serotonin release, sensory neuron activation, and abdominal pain in irritable bowel syndrome. *The American Journal of Gastroenterology*.

[13] Mahjoub FE, Farahmand F, Pourpak Z, Asefi H, Amini Z (2009). Mast cell gastritis: children complaining of chronic abdominal pain with histologically normal gastric mucosal biopsies except for increase in mast cells, proposing a new entity. *Diagnostic Pathology*.

[14] Taylor TJ, Youssef NN, Shankar R, Kleiner DE, Henderson WA (2010). The association of mast cells and serotonin in children with chronic abdominal pain of unknown etiology. *Biomed Central Research Notes*.

[15] Park JH, Rhee PL, Kim HS (2006). Mucosal mast cell counts correlate with visceral hypersensitivity in patients with diarrhea predominant irritable bowel syndrome. *Journal of Gastroenterologa and Hepatology*.

[16] Moore RA, Derry S, Aldington D, Cole P, Wiffen PJ (2012). Amitriptyline for neuropathic pain and fibromyalgia in adults. *Cochrane Database of Systematic Reviews*.

[17] Finnerup NB, Baastrup C (2012). Spinal cord injury pain: mechanisms and management. *Current Pain and Headache Reports*.

[18] Thakur R, Philip AG (2012). Chronic pain perspectives: treating herpes zoster and postherpetic neuralgia: an evidence-based approach. *The Journal of Family Practice*.

[19] Nalamachu S, Morley-Forster P (2012). Diagnosing and managing postherpetic neuralgia. *Drugs and Aging*.

[20] Richards BL, Whittle SL, van der Heijde DM, Buchbinder R (2012). The efficacy and safety of antidepressants in inflammatory arthritis: a Cochrane systematic review. *The Journal of Rheumatology*.

[21] Calandre EP, Rico-Villademoros F (2012). The role of antipsychotics in the management of fibromyalgia. *CNS Drugs*.

[22] Ferjan I, Erjavec F (1996). Changes in histamine and serotonin secretion from rat peritoneal mast cells caused by antidepressants. *Inflammation Research*.

[23] Ferjan I, Erjavec F (1995). Contribution of serotonin reuptake to differential histamine and serotonin secretion from rat peritoneal mast cells. *Inflammation Research*.

[24] Erjavec F, Ferjan I (1991). Characteristics of histamine and serotonin release from rat mast cells induced by thymic peptides. *Agents and Actions*.

[25] Phillips K, Clauw DJ (2011). Central pain mechanisms in chronic pain states—maybe it is all in their head. *Best Practice and Research. Clinical Rheumatology*.

[26] Barbara G, Stanghellini V, De Giorgio R (2004). Activated mast cells in proximity to colonic nerves correlate with abdominal pain in irritable bowel syndrome. *Gastroenterology*.

[27] Crowell MD (2004). Role of serotonin in the pathophysiology of the irritable bowel syndrome. *British Journal of Pharmacology*.

[28] Barbara G, Wang B, Stanghellini V (2007). Mast cell-dependent excitation of visceral-nociceptive sensory neurons in irritable bowel syndrome. *Gastroenterology*.

[29] Costedio MM, Hyman N, Mawe GM (2007). Serotonin and its role in colonic function and in gastrointestinal disorders. *Diseases of the Colon and Rectum*.

[30] Atkinson W, Lockhart S, Whorwell PJ, Keevil B, Houghton LA (2006). Altered 5-hydroxytryptamine signaling in patients with constipation- and diarrhea-predominant irritable bowel syndrome. *Gastroenterology*.

[31] Houghton LA, Atkinson W, Lockhart S, Whorwell PJ, Keevil B (2007). Sigmoid-colonic motility in health and irritable bowel syndrome: a role for 5-hydroxytryptamine. *Neurogastroenterology and Motility*.

[32] Coates MD, Mahoney CR, Linden DR (2004). Molecular defects in mucosal serotonin content and decreased serotonin reuptake transporter in ulcerative colitis and irritable bowel syndrome. *Gastroenterology*.

[33] Spiller R (2007). Recent advances in understanding the role of serotonin in gastrointestinal motility in functional bowel disorders: alterations in 5-HT signalling and metabolism in human disease. *Neurogastroenterology and Motility*.

[34] Gershon MD, Tack J (2007). The serotonin signaling system: from basic understanding to drug development for functional GI disorders. *Gastroenterology*.

[35] De Giorgio R, Barbara G, Furness JB, Tonini M (2007). Novel therapeutic targets for enteric nervous system disorders. *Trends in Pharmacological Sciences*.

[36] Dunlop SP, Coleman NS, Blackshaw E (2005). Abnormalities of 5-hydroxytryptamine metabolism in irritable bowel syndrome. *Clinical Gastroenterology and Hepatology*.

